# Development and validation of a time and motion guide to assess the costs of prevention and control interventions for nosocomial infections: A Delphi method among experts

**DOI:** 10.1371/journal.pone.0242212

**Published:** 2020-11-12

**Authors:** Eric Tchouaket Nguemeleu, Sandra Boivin, Stephanie Robins, Drissa Sia, Kelley Kilpatrick, Sylvain Brousseau, Bruno Dubreuil, Catherine Larouche, Natasha Parisien

**Affiliations:** 1 Université du Québec en Outaouais, Department of Nursing Research, Saint-Jérôme, QC, Canada; 2 Centre Intégré de Santé et de Services de Sociaux des Laurentides, Direction de la Santé Publique, Saint-Jérôme, Québec, Canada; 3 Ingram School of Nursing, McGill University, Montréal, Québec, Canada; 4 Institut de Cardiologie, Montreal Heart Institute, Montréal, Québec, Canada; 5 Centre Intégré Universitaire de Santé et de Services Sociaux du Saguenay, Lac-Saint-Jean, Québec, Canada; 6 Institut National de Santé Publique du Québec, Québec, Canada; Chinese Academy of Medical Sciences and Peking Union Medical College, CHINA

## Abstract

**Background:**

Nosocomial infections place a heavy burden on patients and healthcare providers and impact health care institutions financially. Reducing nosocomial infections requires an integrated program of prevention and control using key clinical best care practices. No instrument currently exists that measures these practices in terms of personnel time and material costs.

**Objective:**

To develop and validate an instrument that would measure nosocomial infection control and prevention best care practice costs, including estimates of human and material resources.

**Methods:**

An evaluation of the literature identified four practices essential for the control of pathogens: hand hygiene, hygiene and sanitation, screening and additional precaution. To reflect time, materials and products used in these practices, our team developed a time and motion guide. Iterations of the guide were assessed in a Delphi technique; content validity was established using the content validity index and reliability was assessed using Kruskall Wallis one-way ANOVA of rank test.

**Results:**

Two rounds of Delphi review were required; 88% of invited experts completed the assessment. The final version of the guide contains eight dimensions: Identification [83 items]; Personnel [5 items]; Additional Precautions [1 item]; Hand Hygiene [2 items]; Personal Protective Equipment [14 items]; Screening [4 items]; Cleaning and Disinfection of Patient Care Equipment [33 items]; and Hygiene and Sanitation [24 items]. The content validity index obtained for all dimensions was acceptable (> 80%). Experts statistically agreed on six of the eight dimensions.

**Discussion/Conclusion:**

This study developed and validated a new instrument based on expert opinion, the *time and motion* guide, for the systematic assessment of costs relating to the human and material resources used in nosocomial infection prevention and control. This guide will prove useful to measure the intensity of the application of prevention and control measures taken before, during and after outbreak periods or during pandemics such as COVID-19.

## Introduction

Nosocomial infections (NIs), also known as healthcare associated infections (HCAIs), are infections acquired during an episode of care in a healthcare facility [1]. These infections are directly related to care and are considered among the most common and preventable adverse events [2]. NIs place a significant burden on patients and hospital staff as they result in medical complications, prolonged hospital stays, high rates of morbidity and mortality, and reduced quality of life; they also place a significant burden on healthcare systems as they result in extra costs related to the extended hospitalization or readmission of patients, patient’s care-related expenses and costs involved in limiting further contagion [3–7]. Similar to other nations, Canada has not solved the problem of NIs. In 2013, the Public Health Agency of Canada reported that more than 200,000 patients contract a NI each year, resulting in more than 8,000 deaths [8]. In 2016, the Canadian Patient Safety Institute estimated that one NI occurs in every 41 hospitalizations, at an estimated cost of 281 million dollars, or 41% of the total cost of adverse events [9]. In the province of Québec, the Comité des infections nosocomiales du Québec (CINQ) estimated, based on American studies, that the annual number of NIs reached somewhere between 80,000 and 90,000 patients in 2005, with an estimated mortality rate of between 1 and 10% [10].

To address the issue of NIs, in 2004 the Québec Ministry of Health and Social Services (MSSS) implemented a mandatory program for monitoring NIs in all hospitals [11]. Since then, this program has become a mainstay for healthcare institutions in managing risks, quality, and patient safety. The program currently tracks different pathogens including: methicillin-resistant *Staphylococcus aureus* (MRSA), *Clostridium difficile*-associated diarrhea (CDAD), colonization and infection caused by multidrug-resistant bacteria such as vancomycin-resistant enterococci (VRE) and carbapenemase-producing *enterobacteria* (CPGNB). Along with NI tracking, target 22 of the MSSS 2015–2020 action plan, *On the prevention and control of NIs for safe delivery of healthcare in the province of Quebec*, is to “evaluate NIPC [Nosocomial Infection Prevention and Control] measures, taking into account the organizational model, the burden of disease, as well as their clinical and epidemiological impacts” [12].

In order to evaluate costs of core NIPC measures, it is helpful to refer to the framework set out by Resar and colleagues of the United States Institute for Healthcare Improvement (IHI) that describes an adverse-events intervention framework of clinical best practices (CBPs) [13]. The authors propose the implementation of “bundles” of evidence-based CBPs in order to ensure the best possible outcomes for: infection prevention, a reduction of complications and the highest level of quality and safety with regards to health care. This framework previously informed Canadian and Quebec healthcare infection prevention and control strategies as well as their respective safety care campaigns [14–16]. Importantly, bundles are defined by the authors as: “A small set of evidence-based interventions for a defined patient segment/population and care setting that, when implemented together, will result in significantly better outcomes than when implemented individually.” Bundles were conceived from a collaborative effort between the IHI and the Voluntary Hospital Association who established a working group to obtain feedback from clinical experts working in 13 hospital intensive care units. The team also undertook an analysis of the medical evidence surrounding clinical processes that were considered to be both the most costly and potentially harmful. From this, design guidelines included 6 key concepts: a) Bundles must have 3–5 interventions and be clinician endorsed; b) Elements in bundles are relatively independent; c) Bundles are specific to a defined patient population; d) Multidisciplinary care teams are involved in the development; and e) Bundle elements should be locally customizable, and descriptive in nature and f) Compliance is measured as an all or none measurement.

According to the IHI, the application of bundled care produces better outcomes and has been shown to bring about monetary savings in the millions. This occurs when bundle elements are measured and accurate is obtained data, either confirming or challenging the assumption that care is being reliably delivered.

Bundles of care, however, do not usually evaluate CBPs that are routinely performed to control NIs. Despite this, four essential and overlapping CBPs are commonly associated with all levels of patient care: a) hand hygiene; b) hygiene and sanitation including the cleaning and disinfecting of surfaces and equipment; c) screening on admission and during hospitalization of carriers and patients who are at risk; and d) application of basic and additional precautions. As has been seen with the COVID-19 pandemic, these four CBPs are very important prevention and control practices to reduce NIs in hospitals [17, 18]. Any bundle designed for improved patient outcomes would be expected to include no less than one of these CBPs and potentially all four. To better understand how these transverse CBPs might impact the costs of care and associated patient outcomes, our team searched for a standardized or systematic way to assess costs.

Studies have reviewed costs and cost-effectiveness related to NIPC management in hospitals. Some studies have assessed the costs and cost benefits of a hand hygiene campaign on the reduction of the incidence of NIs, specifically as they related to MRSA [19], while others focused on the direct costs and cost savings of contact isolation precautions [20–23]. The costs of active surveillance of VRE or MRSA have been estimated in regards to: systematic screening, hygiene procedures, wearing gowns, gloves, single use materials, hydroalcoholic solutions, disinfection procedures, and sanitation [24, 25].

To the best of our knowledge, and after a review of the literature, we found no formal systematic instrument or guide exists that captures the costs required to prevent and control NIs using the four CBPs presented above. Specifically, such a guide should capture, in real time, (i) the time healthcare workers spend on any sequence of actions related to the CBPs of NI prevention and control (hand hygiene, wearing protective barrier apparel, cleaning and disinfecting healthcare equipment and the environment, screening (with or without cultures), additional precautions (by contact, droplet or airborne), isolation, education, training and awareness campaigns); (ii) the materials used for these CBPs; (iii) the products required for these CBPs.

## Objective

To fill this gap, this study aimed to develop and validate a time and motion guide that would be used to measure the costs of the four CBPs of NI prevention and control.

### Methods

The research team formed a working group to establish the steps required to build the time and motion guide. It was composed of: nurses (SB, NP, CL) in the field of infection prevention and control; a professional expert in hygiene and sanitation (BD); and researchers with expertise in time and motion studies (KK); nursing administration (SB); public health (DS, ET); and, health economics (ET).

### Literature review and meetings

Between March 2018 and July 2018, a review of the literature was undertaken in order to appraise and synthesize the data on the costs of the four CBPs, as well as the human resources, material and product costs related to NI prevention and control. We searched the scientific and grey literature using Medline, Cinhal, Pubmed, and Cochrane databases as well as Canadian and Québec government and World Health Organisation (WHO) websites, limiting our search from 2010 to 2018 inclusive. We used the following research strategy and key words: (tool OR instrument OR scale OR measure) AND (“nosocomial infections” OR “hospital acquired infections” OR “health care associated infections”) AND (prevention OR intervention OR treatment OR program OR screening OR assessment OR test OR diagnosis OR “screening tool” OR strategy OR management) AND (cost*). After screening the title and abstract of the articles, we selected and reviewed 22 articles [19–41] and more than ten international and national reports [42–56]. Five meetings of the working group occurred between March and July of 2018. As noted above, the literature was examined to better understand how to measure the costs of CBPs, however a comprehensive cost-accounting tool did not emerge. In order to accurately measure costs, we considered incorporating the methodology from time-driven activity-based costing (TDABC) [57]. TDABC functions by dynamically allocating costs related to the consumption of resources across human-driven processes, with the purpose of adding up costs throughout a supply chain to determine overall expenses. Adapted from a business model, TDABC has been used in healthcare to measure human resource costs related to specific medical conditions such as paediatric surgery, ear nose and throat medicine, dermatology and psychiatry [for a review see 58]. For our purposes, instead of measuring a medical condition we focused on our four CBPs and began breaking down each one in granular fashion, considering the start and end of each process. The activity and location of each CBP was determined. Process maps and all resources used for each step of any CBP. This process revealed that each CBP actually had layers of elements that resulted in the emergence of sub dimensions (later defined as items) that were not previously considered.

Further discussions helped to establish models of workflow of different healthcare staff, and brought to light which CBPs might be used to accomplish individual tasks. In order to capture the time required for CBPs, it was proposed that a chronometer be integrated that could measure at least one, but preferably more than one simultaneously occurring action. This proposal was accepted, and it was decided that external observers would use the guide and chronometer to capture data continuously. These two decisions fall in line with a recent schema of time and motion studies [see 59 for a review]. Members of the team proposed that this detailed and direct continuous observation approach to data collection may result in the tendency of staff to improve their performance [for an example in healthcare see 60]. This point was discussed, and accepted as a limitation of the instrument. Sessions were iterative, with issues being revisited as the guide was developed. By July of 2018, these sessions had proven to be key in helping to establish a consensus between all members as to the necessary components of an algorithm that would serve as a framework for the time and motion guide.

### Creation of an algorithm and time and motion guide

**Fig 1:**
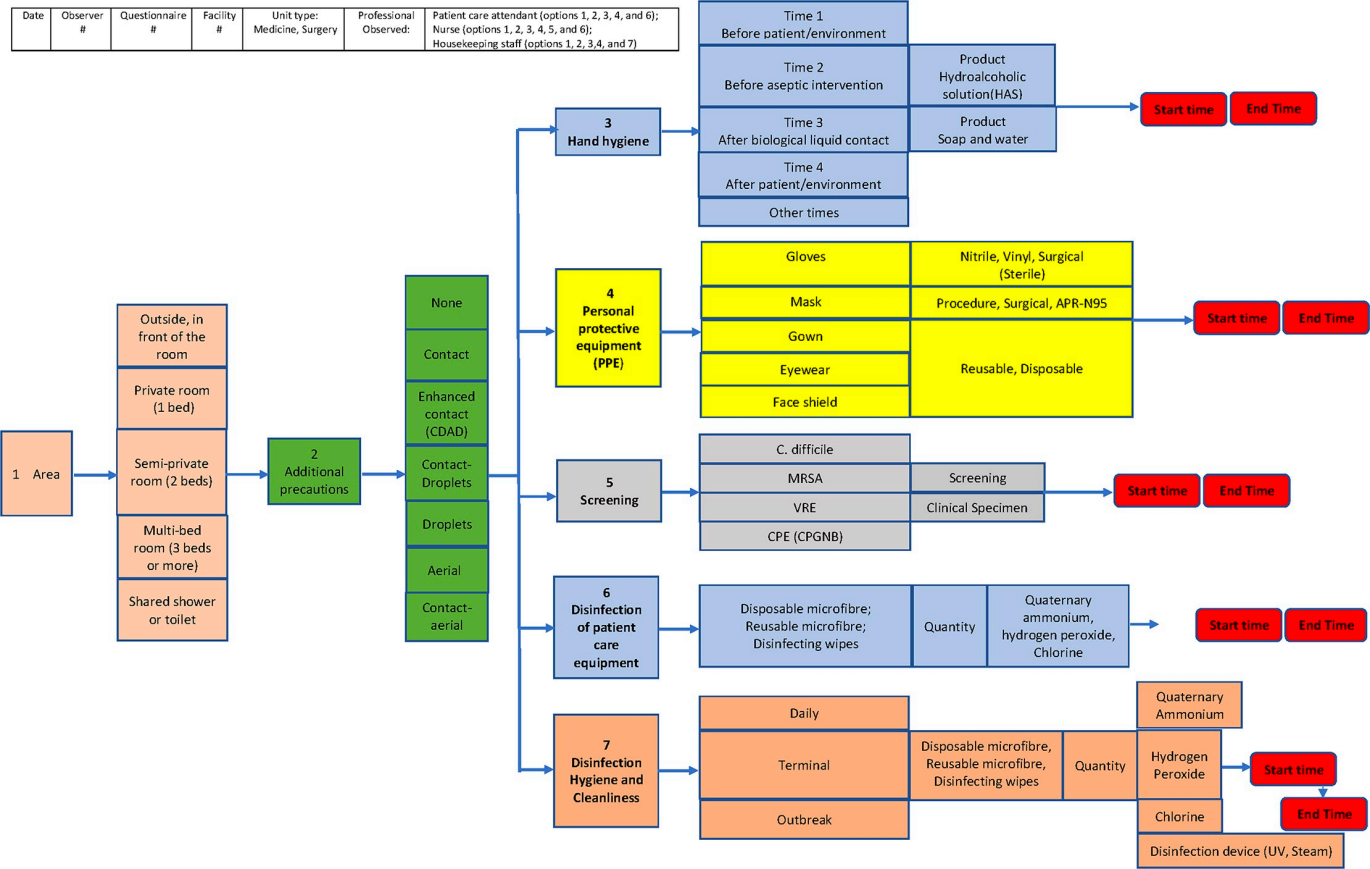
Time and motion guide algorithm.

To inform our process, the working group first drafted an initial version of the time and motion algorithm (see Fig 1). The algorithm outlines a series of sequential steps observers would follow when recording the time necessary for CBPs and for the materials and products used in daily prevention and control of NIs. The algorithm first included observer and location-specific measures to be captured including: identification of the observer; the date and site where measurements would be taken; the hospital unit (medicine or surgery), and the title of the professional being observed (patient-care attendant, nurse or housekeeping staff). The algorithm was further divided into seven main dimensions: 1) area being evaluated, 2) hand hygiene, 3) personal protective equipment, 4) screening, 5) disinfection of patient care equipment, 6) overall disinfection, hygiene and cleanliness, and 7) additional precautions. Each dimension contained sub-categories of items that described, in a more granular fashion, the time and/or material to capture. This information would provide a more precise measurement of the amount of personnel time and material costs used, and where these resources were spent. For example, the first sub-category of ‘area’ would clarify where (i.e., a private, semi-private or multi-bed room) staff spent time in NIPC actions. In the category of hand-washing, the sub-categories to identify included: when personnel washed their hands (before, during, or after patient intervention) along with the product they used: soap or hydroalcoholic solution. The final and most essential level of the algorithm notes that for each infection and control opportunity, time taken (in seconds) and products used will be systematically recorded.

The algorithm then helped guide the research team to develop the second phase: the creation of the time and motion guide, effectively operationalizing the constructs set out in the research question. The time and motion guide contains items in each of the seven dimensions. The guide was created as a paper questionnaire to be used for the standard measurement of NIPC costs. In order to optimally integrate the chronometer, the questionnaire was incorporated into an online application using the software platform Dataformz (https://www.dataformz.com/en/). This digital version of the questionnaire allows for access from a variety of devices (tablet, phone or computer) in order to capture all actions in real time. It also provides a tool that facilitates nurse observer training and pilot testing province-wide. Users access the Dataformz platform using a secure URL and identify themselves by choosing their name from a list of user names. The dashboard first displays information that is deemed essential, such as the health care facility location, unit, personnel job title, and area being assessed; here users are presented with mandatory fields. At the completion of these fields, users are prompted to ‘submit’ their answers, effectively registering the collected data for that individual in the online database. The nurse observer following this professional then opens pages of the app where appropriate categories of the CBP are measured. For time measurements, users make use of the online chronometer to quantify movements in real time, and this, even if two or more actions are underway, as overlapping actions can be recorded. Recordings of time can be saved or, if there was an error, discarded. Similarly, all materials used in NIPC are listed on the platform as radio buttons or check-all boxes to facilitate data collection. Open text boxes (‘other’) are also provided in sections of the app where the description of a product is not predetermined. When an observation is complete, the user clicks the ‘end questionnaire’ button to submit their record.

Finally, a user manual of the guide was developed to facilitate training of nurse observers and ensure all observations are entered in a standardized fashion. The manual provides a description of the research team and a brief summary of the study’s purpose; the time and motion algorithm is annexed for reference purposes. The user manual describes in detail how to navigate each page of the online platform and how to correctly input observations. For each category of the guide, written explanations are accompanied by screenshots of the interface that users should expect to encounter on the app.

### Construction of the time and motion guide questionnaire

The time and motion guide questionnaire to be sent to Delphi panelists included an initial version with 9 dimensions: (1) Identification [11 items]; (2) Personnel [4 items]; (3) Zone [2 items]; (4) Additional Precautions [1 item]; (5) Hand Hygiene [2 items]; (6) Personal Protective Equipment [2 items]; (7) Screening [4 items]; (8) Cleaning and Disinfection of Patient Care Equipment [5 items]; (9) Hygiene and sanitation [5 items]. The questionnaire was designed to elicit both qualitative and quantitative responses for the guide items and dimensions, and to obtain a qualitative evaluation of the web app functioning. Panelists were asked to rate all items in all dimensions of the guide, using a 4-point Likert scale from 1 = “*not relevant*” to 4 = “*very relevant without corrections*” as shorter Likert scales have produced stable findings in Delphi studies previously [61, 62]. Panelists were also provided an open-ended question about each item’s relevance, should they have a suggestion for improvement or other opinion.

### Content validity of time motion guide

The content validity was assessed using the Delphi Technique among experts. The Delphi Technique is a well described group survey technique widely used in health research that results in the narrowing of a consensus on a topic of public importance [63]. The Delphi process allows for a variety of methodological approaches, including qualitative and quantitative data gathering and analysis that can be revised. It also allows for the investigation of a solution that may not yet exist. Due to its anonymity and iterative process, the Delphi method is considered to be less prone to bias as expert participants have the opportunity to provide their honest opinion or creative solution without feeling the need to agree with dominant personalities [64].

**Fig 2:**
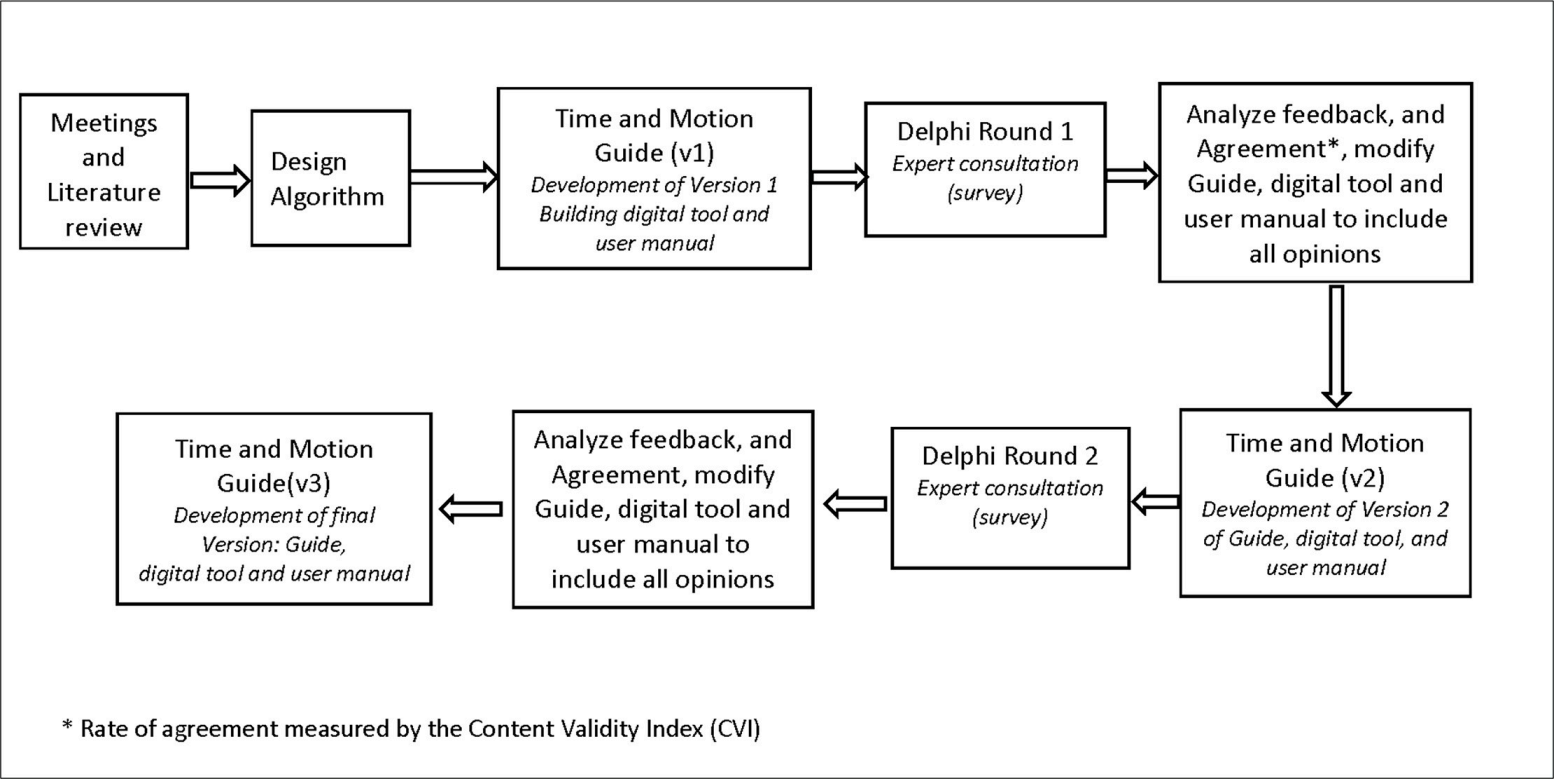
Validation process of the time and motion guide using the Delphi technique.

Our team used this systematic and interactive forecasting method as has been done by others in the field of health care [65–67], to obtain consensus from a panel of experts, consulted over two or more rounds, for feedback on the time and motion guide. Experts were invited to two successive rounds of review. After each round, quantitative and qualitative data collected were analysed in order to assess the agreement and consensus. In each step, the panelists received the time and motion guide questionnaire, access to the Dataformz website and the user manual for revision and resubmission. Prior to the second round, panelists received their own feedback along with anonymized qualitative feedback from others (see S1 File for the final questionnaire provided to experts). Once two rounds were complete, the working group addressed any remaining discrepancies and produced a final version of all tools for pilot testing. Fig 2 presents the Delphi process for this study.

### Delphi technique: Selection of expert panel and procedure

In order to consider a variety of opinions from participants having lived and/or professional experience in NI prevention and control, 18 experts [as recommended by 68] from two distinct viewpoints were selected: those who incorporated NIPC measures directly in their workday and those who could be considered content experts as a result of their professional training. The first group (12 experts) was composed of professionals that worked directly in infection prevention and control and were considered to have experiential expertise that complemented their professional training. A group of six was chosen from two healthcare facilities that differed in their structure, one being associated with a university and thus serving as a teaching facility, the other not. The time motion guide was analyzed by: a manager in charge of NIPC, two nurses, a microbiologist/infectious disease expert, and two health and sanitation staff. The second group of experts (n = 6) were chosen for their knowledge and expertise in the field, their active involvement in infection prevention and control via their role in government and public policy implementation, or because they were key opinion leaders. This group was made up of two microbiologist/infectious disease experts, two clinical nurse specialists in NIPC and two hygiene and sanitation advisors. We purposely sourced male and female panelists from different geographic regions of Quebec, Canada, to ensure the feedback would be valid for, and representative of, practices across the province. All experts who expressed interest in the study (n = 18) were sent a consent form to be returned by e-mail and all then provided informed consent.

The two rounds of the Delphi survey were conducted between July 2018 and January 2019. All experts were sent the time and motion guide questionnaire and asked to rate each item and dimension between 1 *“not relevant”*, 2 *“relevant with major corrections”*, 3 *“relevant with few corrections”*, and 4 *“very relevant without corrections”*, based on its relevance for the assessment of costs related to the four CBPs. The content validity was assessed using the content validity index (CVI) [69], which was set as a percentage, counting each item and dimension rated *4 “very relevant without corrections”* over the total number of replies by experts for the item or dimension.

Three levels of review were considered to assess the content validity or agreement between experts. First, when the CVI for a dimension (CVI_D) was less than 0.80 (80%), the dimension was considered not acceptable. The dimension was then completely reviewed and revised according to the proposed corrections. The resultant items and dimension were then resubmitted to the same experts in the next Delphi round. When the CVI_D was equal to or greater than 0.80, the dimension was considered acceptable. Second, the CVI for individual items (CVI_I) of all dimensions was assessed. As above, when CVI_I was less than 0.80, the item was considered not acceptable and revised according to the proposed corrections. The item was then resubmitted to the same experts in the second round. When the CVI_I was equal to or greater than 0.80, the item was kept. In a third and final level of review, items that were revised but did not improve to the level of 0.80 following two rounds of Delphi review were revisited by the working group in order to finalize the items and address concerns expressed by the experts in round two.

### Reliability of time motion guide

Finally, in order to ascertain reliability, (i.e., how much of the variance in observed ratings was due to a true difference in score, and not measurement error between groups of expert panelists), we performed non-parametric Kruskall-Wallis ANOVA of rank tests for each dimension, with 5% threshold, to demonstrate any effect of group. When the Kruskall-Wallis tests of a dimension was not statistically significant, it meant that all the responses were acceptably constant between the panel experts, or reliable. Otherwise, the dimension and its items were revisited in its entirety and revised according to the proposed corrections by experts. The dimension would then be resubmitted to the same experts in another round. All the revisions were discussed and validated by the research team during working group meetings. Data were analysed using SPSS version 26 (Armonk, NY, USA: IBM Corp). Ethical approval was obtained from the Centre intégré de santé et services sociaux des Laurentides (reference #: 2017MP-364-É and MP-28-2018-002) and the Université du Québec en Outaouais. All participants in the Delphi panel provided written informed consent.

## Results

### Delphi: Rounds 1 & 2

In an initial round, all 18 experts were invited to complete the time and motion survey using the first version of the algorithm, user manual and web platform; tools were provided in French versions as all panelists were Francophones. Of these, 16 experts (88.9%) completed the questionnaire: 6 from the non-university affiliated hospital (group 1), 5 from the university-affiliated hospital (group 2), and 5 content experts (group 3). For all response rates, see Table 1. For quantitative responses, only one dimension of the guide, (*cleaning and disinfection of patient care equipment*) met our CVI_D acceptable limit of greater than 80%, at 83.4%. However, only two items within this dimension met a CVI_I of 80%. Reliability for all dimensions was acceptable (i.e., the groups did not significantly differ) with the exception of the dimension *hygiene and sanitation*, where the effect of expert group was significant, X^2^(2) = 9.02 p = 0.01. Thus, all the dimensions were reviewed and analyzed based on the comments and suggestions of experts. The guide was reworked and the questionnaire adapted for round 2, with all anonymized responses from the first round provided to experts.

**Table 1:** Delphi round 1 content validity index for all dimensions and items and panelist reliability by site.

DIMENSIONS AND ITEMS	CVI	Kruskal Wallis test	
		Value	P-value
**Identification of Facility**	**75.4%**	**2.01**	**0.37**
Identification of Health Care Facility	93.8%		
Number of beds in the facility	81.3%		
Type of Unit	66.7%		
Number of beds in the unit	62.5%		
Format of hydroalcoholic solution in unit	68.8%		
Brand of hydroalcoholic solution in unit	80.0%		
Brand of soap used in unit	75.0%		
**Personnel Identification**	**76.6%**	**3.12**	**0.21**
Health care professional observed	87.5%		
Level of education	56.3%		
Number of years of experience in hospital unit	81.3%		
Number of years of experience in position held	81.3%		
**Zone**	**66.7%**	**0.09**	**0.96**
Area where action is being observed	81.3%		
Outside of room	50.0%		
Room	68.8%		
**Additional Precautions**	**66.7%**	**0.02**	**0.37**
Type of additional precautions	75.0%		
**Hand Hygiene**	**62.5%**	**2.80**	**0.25**
Moment	56.3%		
Products used	68.8%		
**Personal protective equipment**	**78.1%**	**4.33**	**0.12**
Action of donning or removing equipment	81.3%		
Equipment used	75.0%		
**Screening**	**56.3%**	**1.73**	**0.42**
C.*difficile*	61.5%		
Number of samples (swabs) used for MRSA	54.5%		
Number of samples (swabs) used for VRE	54.5%		
Number of samples (swabs) used for CPGNB	54.5%		
**Cleaning and disinfecting of small equipment**	**83.4%**	**1.88**	**0.39**
Type of health care equipment	68.8%		
Product used	93.3%		
Quantity of each product used	100.0%		
Products	71.4%		
**Hygiene and Sanitation**	**70.0%**	**9.02**	**0.01**
Kind of cleaning	62.5%		
Zone cleaned	68.8%		
Materials used	75.0%		
Products	81.3%		
Quantity of product	62.5%		
**OVERALL**	**70.6%**	** **	** **

In round 2, all 16 panelists who had completed round 1 were invited to respond to the second version of the questionnaire in order to evaluate the guide using the user manual and web platform. In this round, 14 experts (87.5%) completed the survey with 5 responses each from the university and non-university site, and 4 from content experts. Content validity improved markedly on round 2, with all dimensions surpassing the threshold of 80% (see Table 2). However, one item each from the dimension *Identification* and *Zone* as well as three within the dimension of *Hygiene and sanitation*, had CVIs of below 80%, and were flagged for further review. In this second round, reliability was acceptable for all dimensions except two. In *Zone*, the Kruskall Wallis test revealed a significant difference between groups of experts: X^2^(2) = 10.20 p = 0.006, as it did in the dimension *Hygiene and Sanitation*, X^2^(2) = 9.95 p = 0.007. Comments were reviewed in order to determine the source of disagreement.

**Table 2:** Delphi round 2 content validity index for all dimensions and items and panelist reliability by site.

DIMENSIONS AND ITEMS	CVI	Kruskal Wallis test	
		Value	P-value
**Identification of Facility**	**93.5%**	**0.208**	**0.901**
Identification of Health Care Facility	100.0%		
Number of beds in the facility (as per permit)	100.0%		
Unit name	100.0%		
Type of Unit	100.0%		
Number of beds in the unit (as per permit)	100.0%		
Number of hydroalcoholic solution (HAS) dispensers in corridor and at door to room, in unit	85.7%		
Format of hydroalcoholic solution in unit	100.0%		
Brand of hydroalcoholic solution used in unit	100.0%		
Brand of soap used in unit	100.0%		
Brand of the 1st hygiene and sanitation product with diluent	64.3%		
Brand of the 1st hygiene and sanitation product ready to use	78.6%		
Use of hygienic covers (yes-no)	92.9%		
**Personnel Identification**	**97.1%**	**1.086**	**0.581**
Identification of Health Care Facility	100.0%		
Unit name	100.0%		
Code of the professional being observed	85.7%		
Health care professional observed	100.0%		
Number of years of experience in current position	100.0%		
**Zone**	**88.1%**	**10.202**	**0.006**
Area where action is being observed	85.7%		
Outside of room (without corridor)	85.7%		
Corridor	71.4%		
Private room with 1 bed	92.9%		
Semi-private with 2 beds	92.9%		
Room with 3 beds	92.9%		
Room with 4 beds	92.9%		
Room with 5 beds	92.9%		
Room with 6 beds	92.9%		
Other area (describe)	92.9%		
Number of HAS (hydroalcoholic solution dispensers) in private room	85.7%		
Number of HAS dispensers in semi-private room (2 beds)	85.7%		
Number of HAS dispensers in room with 3 beds	85.7%		
Number of HAS dispensers in room with 4 beds	85.7%		
Number of HAS dispensers in room with 5 beds	85.7%		
Number of HAS dispensers in room with 6 beds	85.7%		
Number of HAS dispensers in room, other area	85.7%		
**Additional Precautions**	**85.7%**	**2.000**	**0.368**
Type of precautions	85.7%		
**Hand Hygiene**	**100.0%**	**0.000**	**1.000**
Moment	100.0%		
Products used	100.0%		
**Personal protective equipment**	**89.3%**	**2.333**	**0.311**
Action of donning or removing equipment	85.7%		
Equipment used	92.9%		
**Screening**	**90.8%**	**4.281**	**0.124**
*C*.*difficile*	100.0%		
MRSA-→note site of sample	85.7%		
Number of samples (swabs) used for MRSA	92.9%		
VRE →note site of sample	85.7%		
Number of samples (swabs) used for VRE	92.9%		
CPGNB→ note site of sample	85.7%		
Number of samples (swabs) used for CPGNB	92.9%		
**Cleaning and disinfecting of small equipment**	**94.6%**	**5.500**	**0.064**
Type of health care equipment	92.9%		
Product used	92.9%		
Quantity of each product used	100.0%		
Products	92.9%		
**Hygiene and Sanitation**	**80.4%**	**9.952**	**0.007**
Kind of cleaning	78.6%		
If terminal, additional precautions	78.6%		
Materials used	78.6%		
Products	85.7%		
**OVERALL**	**91.1%**		

### Description of time and motion guide (final version)

The final version of the guide contains eight of the nine original dimensions, with the dimension Zone merged with *Hygiene and Sanitation*. These eight dimensions include: Identification [83 items]; Personnel [5 items]; Additional Precautions [1 item]; Hand Hygiene [2 items]; Personal Protective Equipment [14 items]; Screening [4 items]; Cleaning and Disinfection of Patient Care Equipment [33 items]; and Hygiene and Sanitation [24 items].

The guide and online platform have been substantially adapted. For example, the first section, *Identification*, was expanded from 12 items in round 2, to 83 items in the final version. This increased level of detail provides the user with the ability to measure items such as the brand and format of hydroalcoholic solution being used (gel or foam) and the count of disposable items. It also includes newly considered items that have a profound impact on NICP costs, such as staff attendance at NIPC training and information sessions as well as awareness campaigns. Other sections that were vastly revised included both sections that enumerated equipment (either personal protective equipment or patient monitoring equipment), to include detailed lists of all equipment used. The guide includes chronometers at the level of all items that require personnel time; the digital stopwatch can be set to measure time, either sequentially or simultaneously with CBPs, as needed. The complete paper version of the guide can be seen in S2 File. All changes to the time and motion guide were implemented in the final version of the web app and user manual in preparation for pilot testing.

## Discussion

The goal of this study was to develop and validate a time and motion guide to facilitate the objective assessment of the costs of four CBPs of NI prevention and control, using a systematic approach. Our process first defined an original set of items chosen from an extensive review of the literature. These were later expanded upon to create a final version of the time and motion guide in three successive waves of review, two of these undertaken by a panel of experts. By using the Delphi technique, preliminary evidence of validity and reliability of the guide were gathered.

Content validity is an important step in the development of questionnaires and quantifies the level of agreement between experts; we decided *a priori* to evaluate the assessments of our panelists using the CVI, both at the item and dimension level. According to Polit [69] the index is useful in questionnaire development as it provides *“*a *focus on consensus rather than consistency estimates*, *ease of computation*, *understandability and ease of communication*, *provision of both item diagnostic information and scale validity information”*. By the end of two rounds of Delphi review, the CVI was deemed acceptable in all dimensions, and only failed to meet the 80% cut-off for six individual items. The Kruskall Wallis tests of reliability revealed disagreement between the groups in two dimensions: *Zone*, and *Hygiene and Sanitation*.

However, divergent opinions are an expected outcome of the Delphi process, and have been considered as representing smaller groups who hold a “clustered consensus” within the panel [70]. Therefore, along with the commentary provided by the panelists these items and dimensions were conceptually reviewed and extensively modified. The dimension of *Zone* was merged within *Hygiene and Sanitation*. This change reflected not only the poor statistical reliability between expert groups but also the context of sanitation procedures and how they might differ in terms of time and intensity, between physical spaces in clinical settings. For example, the cleaning of an isolation room may require more time or products as all surfaces and equipment are meticulously disinfected or discarded when patients are discharged. This intense terminal cleaning is not comparable to the hygiene and sanitation practices in a common area such as a hallway, where floors and hand railings receive daily disinfection.

The Delphi technique can provide invaluable insight for the development of new solutions; this is especially true within evolving disciplines such as healthcare. For example, Njuangang and colleagues [71], via the Delphi technique, established 11 novel critical success factors and performance measures within hospital maintenance to reduce the risk of maintenance-associated NIs. The ability to identify salient performance measures is also reflected in our study; over two rounds of review, 16 experts expanded and enriched the guide’s functionality and precision to create a tool with 8 dimensions and over 175 items that would assess cost. The strength of our process rested in part on our choice of panelists- a heterogeneous mix of stakeholders who were wholly invested in the innovation of the guide, from its development to its indicators of credibility and quality. From the original group of 18 invitees, 16 members completed the first wave of review, and all but two members completed the second. Those who recused themselves joined our research team in an advisory capacity to further the development and pilot testing of the guide, thus providing expert feedback as part of the working group in the final iteration.

### Implications for research and practice

The time and motion guide, in its current version, is an instrument that can effectively allow health care managers and clinicians to assess the costs of nosocomial infection prevention and control practices. This information will complement epidemiological data that tracks the incidence of infection in health care facilities. Importantly, the guide provides a way to measure the cost effectiveness of CBPs in relation to the pathology of NIs. At the time of writing of this manuscript, measuring cost effectiveness in relation to infection control is exceptionally fitting as the world is struggling with the COVID-19 pandemic, where unprecedented infection control and prevention measures are being undertaken.

Aside from cost estimates, the time and motion guide is strategically detailed and can easily be implemented as an online instrument to serve as a training tool for personnel involved in NI control. Along with enumerating, for the user, materials and products used to control the spread of infection, the chronometer of the guide provides an objective measurement of the time taken for CBPs, such as hand washing. While subjectively, healthcare staff may consider their hand hygiene techniques adequate, they may not meet hand hygiene guidelines set out by the WHO that suggest 20–30 seconds for hydro-alcoholic solutions, or 40–60 seconds for soap and water [56]. It could thus serve in training and continuing education. The guide could also help us to better understand how personnel adapt their clinical best care practices to their working conditions. An analysis of the intensity of nosocomial prevention and control measures in terms of costs (time, materials and products) could be undertaken to compare practices and their related costs before, during and after outbreaks or pandemic events such as COVID-19.

### Strengths and limitations

A strength of this study is that the time and motion guide represents a new and completely original approach to nosocomial infection prevention and control cost assessment. By adapting the questionnaire to a web-based application, the guide can span geographic separation of health care facilities where it can be tested, and may deliver a framework upon which to structure other health care associated costing tools. The time motion guide is innovative in that it simultaneously measures more than one action at a time, compared to other time motion guides that normally only measure a single action at a time [72, 73]. A further strength of our process is that the Delphi technique was assiduously conceived, administered and reported, an underlying prerequisite for credible conclusions. We attempted to fulfill all sixteen recommendations set out in the Conducting and REporting of DElphi Studies (CREDES) framework [63]. However, this study has limitations. The acceptability of the time and motion guide, and feasibility of use, will have to be pilot-tested by users in different health care facilities and in real-life settings under normal work conditions. Only then will the efficiency and effectiveness of the guide become evident. The study has certain other limitations, notably, that our guide is based on a health care system in one province and country. Health care settings, NI rates and NIPC practices will differ between developed and developing nations, limiting the generalizability of the guide.

## Conclusion

We developed and validated a time and motion guide for the measurement of costs of clinical best practices used for nosocomial infection control and prevention in medical and surgical hospital units. Future research should include pilot testing of the guide with a view to how the instrument could be adapted for use in other healthcare settings.

## Supporting information

S1 File Time motion guide questionnaire for experts. (DOCX)

S2 File Time motion guide paper version. (DOCX)

S3 File CVI and reliability dataset. (XLSX)

## Abbreviations

BD: Bruno Dubreuil
CBP: Clinical best practice
CDAD: Clostridium difficile associated diarrhea
CINQ: Comité des infections nosocomiales du Québec
CISSS: Integrated Health and Social Services Centre for the Laurentian region, Quebec
CIUSSS: Integrated University Centre for Health and Social Services
CL: Catherine Larouche
CPGNB: Carbapenemase-producing Enterobacteriaceae
CVI: Content validity index
DS: Drissa Sia
ET: Eric Tchouaket
KK: Kelley Kilpatrick
MRSA: Methicillin-resistant Staphylococcus aureus
MSSS: Quebec’s Ministry of Health and Social Services
NI: Nosocomial infection
NIPC: NI Program for Prevention and Control
NP: Natasha Parisien
SB: Sandra Boivin
SBR: Sylvain Brousseau
SR: Stephanie Robins
SPIN: Quebec’s Provincial NI Surveillance Group
TDABC: Time driven activity based costing
VRE: Vancomycin-resistant enterococcus
