# Supplementary material for: Development and validation of a time and motion guide to assess the costs of prevention and control interventions for nosocomial infections: A Delphi method among experts
Source: PLoS One. 2020 Nov 12;15(11):e0242212. doi: 10.1371/journal.pone.0242212 (PMC7660509; doi:10.1371/journal.pone.0242212)
Supplement: S1 File — (DOCX) [file pone.0242212.s001.docx]

**Form for evaluating the relevance of dimensions and items of the time and motion guide *@nePCI***

Using the instruction manual please indicate with a checkmark whether the dimensions and individual items are:

not relevant (1),

not very relevant with major correction (2),

relevant with few corrections (3),

very relevant without corrections (4).

You must, in the column "Comments on the relevance of the dimension”, tell us why the dimension or item would be irrelevant and what improvements or corrections should be made.

In the last column "Comments related to the operation of the dimension in the application", please use this space for comments related to the web application @nePCI function for data collection related to each section.

| **Dimension** | **Relevance of dimensions** | | | | **Comments on the relevance of the dimension** | | | **Comments on the**  **dimension’s function in the web application** |
| --- | --- | --- | --- | --- | --- | --- | --- | --- |
|  | 1  not relevant | 2  not very relevant with major corrections | 3  relevant with few corrections | 4  very relevant without corrections |  | | |  |
| **Identification** |  | | | | | | | |
| Identification of Health Care Facility |  |  |  |  |  | | |  |
| Number of beds in the facility (as per permit) |  |  |  |  |  | | |  |
| Name of Unit |  |  |  |  |  | | |  |
| Unit type  Medicine  Surgery  Other Unit |  |  |  |  |  | | |  |
| Number of beds in unit  (as per permit) |  |  |  |  |  | | |  |
| Number of Hydroalcoholic solution (HAS) dispensers:  in the corridors and  at doors of rooms in unit |  |  |  |  |  | | |  |
| Format of HAS solution in unit:  Foam  Liquid/Gel |  |  |  |  |  | | |  |
| Brand of HAS solution in unit |  |  |  |  |  | | |  |
| Brand of soap used in unit |  |  |  |  |  | | |  |
| Name (Brand) of the 1st hygiene and sanitation product with dosing device (diluent) |  |  |  |  |  | | |  |
| Name (Brand) of the 1st ready-to-use hygiene and sanitation product |  |  |  |  |  | | |  |
| Use of hygienic covers:  Yes  No |  |  |  |  |  | | |  |
| **Identification** |  | | | | | | | |
| Identification of Health Care Facility |  |  |  |  | |  |  | |
| Name of Unit |  |  |  |  | |  |  | |
| Code assigned to person being observed |  |  |  |  | |  |  | |
| Healthcare Professional being observed:  Auxiliary Nurse  Nurse (College trained)*  Nurse (BSc.)  Clinical Nurse Specialist  General Practitioner  Medical Specialist  Orderly  Hygiene and sanitation worker |  |  |  |  | |  |  | |
| Number of years of experience in current position  0-1 year  1-5 years  5-10 years  10 years and over |  |  |  |  | |  |  | |
| **Zone** |  | | | | | | | |
| Location where action is being observed:  Outside of room  Inside of room |  |  |  |  | |  |  | |
| Outside of Room:  Corridor  Toilet for visitors/patients in hallway  Shower for patients in hallway  Other (describe) |  |  |  |  | |  |  | |
| If Corridor, choose :  Hand rail  Hand hygiene station |  |  |  |  | |  |  | |
| Room:  Private room (1 bed)  Semi-private (2 beds)  Room with 3 beds  Room with 4 beds  Room with 5 beds  Room with 6 beds  Other (describe) |  |  |  |  | |  |  | |
| Number of HAS dispensers in private room  0-1-2-3 |  |  |  |  | |  |  | |
| Number of HAS dispensers in  semi-private room (2 beds)  0-1-2-3-4 |  |  |  |  | |  |  | |
| Number of HAS dispensers in  room with 3 beds  0-1-2-3-4-5 |  |  |  |  | |  |  | |
| Number of HAS dispensers in  room with 4 beds  0-1-2-3-4-5-6 |  |  |  |  | |  |  | |
| Number of HAS dispensers in  room with 5 beds  0-1-2-3-4-5-6-7 |  |  |  |  | |  |  | |
| Number of HAS dispensers in  room with 6 beds  0-1-2-3-4-5-6-7-8 |  |  |  |  | |  |  | |
| Other Room:  Number of HAS dispensers in room  0-1-2-3-4-5-6-7-8-9-10 |  |  |  |  | |  |  | |
| **Additional Precautions** |  | | | | | | | |
| Type of Additional precautions  Contact  Droplet  Droplet - contact  Airborne  Airborne - contact  Other |  |  |  |  | |  |  | |
| **Hand Hygiene** |  |  |  |  | |  |  | |
| Moment: choose the moment that corresponds to the beginning of the action  Before coming in contact with the patient or their environment  Before an aseptic procedure  After handing body fluids or after removal of gloves  After coming in contact with the patient or their environment  Other (describe) |  |  |  |  | |  |  | |
| Products used  Soap and water  Hydroalcoholic solution on table (500 ml)  Hydroalcoholic solution on wall (1L)  Hydroalcoholic solution pocket size (45-50 ml) |  |  |  |  | |  |  | |
| **Personal protective equipment (PPE)** |  | | | | | | | |
| Action of donning or removing equipment  Donning  Removing |  |  |  |  | |  |  | |
| Equipment Used  Nitrile gloves  Vinyl gloves  Sterile gloves  Procedural or surgical mask  N95 Mask  Disposable gown  Reusable gown  Disposable glasses  Reusable glasses  Disposable visor  Reusable visor  Other (describe) |  |  |  |  | |  |  | |
| **Screening** |  |  |  |  | |  |  | |
| Sample  *C. difficile* (clinical specimen)  MRSA (screening)  VRE (screening)  CPGNB (screening) |  |  |  |  | |  |  | |
| For MRSA, note site of sample  Groin and armpits  Colostomy  Nostrils  Wound(s)  Tracheostomy  Urine  Other (describe) |  |  |  |  | |  |  | |
| Number of samples (swabs) used for MRSA  1-2-3-4-5-6-7-8-9-10 |  |  |  |  | |  |  | |
| For VRE, note site of sample  Colostomy  Wound(s)  Rectum  Tracheostomy  Urine  Other (describe) |  |  |  |  | |  |  | |
| Number of samples (swabs) used for VRE used*  1-2-3-4-5-6-7-8-9-10 |  |  |  |  | |  |  | |
| For CPGNB note site of sample:  Groin and armpits  Colostomy  Wound(s)  Rectum  Tracheostomy  Urine  Other (describe) |  |  |  |  | |  |  | |
| Number of samples (swabs) used for CPGNB used*  1-2-3-4-5-6-7-8-9-10 |  |  |  |  | |  |  | |
| **Cleaning and disinfection of small healthcare equipment** |  | | | | | | | |
| Type of health care equipment:  Multifunction vital signs monitor  Bassin  Commode chair  Wheelchair  Stretcher  Blood glucose monitor  Walker  Positive displacement pump  Syringe pump  Pulse oximeter  Portable bladder scanner  Stethoscope  Measuring cup  Blood pressure monitor  Thermometer  I.V. Stand  Urinal  Other(describe)___________ |  |  |  |  | |  |  | |
| Material Used  Reusable cloths (washcloths, cotton cloths, etc.)  Microfiber cloths  Disinfectant disposable wipes  Alcohol swabs  Other(describe)________ |  |  |  |  | |  |  | |
| Quantity (number) of materials used  1-2-3-4-5-6-7-8-9-10 |  |  |  |  | |  |  | |
| Products  Quaternary ammonium  Hydrogen peroxide  Chlorine solution  Alcohol  Other(describe)________ |  |  |  |  | |  |  | |
| **Hygiene and Sanitation** |  | | | | | | | |
| Kind of cleaning  Daily regular  Daily additional precautions  Terminal additional precautions  Terminal discharge /transfer  Outbreak (high touch surfaces) |  |  |  |  | |  |  | |
| If terminal additional precautions:  One step  Two steps  Three steps |  |  |  |  | |  |  | |
| Material used:  Reusable cloths (washcloths, cotton cloths, etc.)  Disinfectant disposable wipes  Microfiber cloths  Alcohol swabs  Floor buffers  Other(describe)________ |  |  |  |  | |  |  | |
| Products used  Quaternary ammonium ready to use  Quaternary ammonium to dilute  Hydrogen peroxide ready to use  Hydrogen peroxide to dilute  Chlorine solution ready to use  Chlorine solution to dilute  Other(describe)___________ |  |  |  |  | |  |  | |

* = In Quebec, Canada, nurses are also trained at the college level (*Diplôme d'études collégiales*)
